# Second-order optimality conditions for nonlinear programs and mathematical programs

**DOI:** 10.1186/s13660-017-1487-8

**Published:** 2017-09-08

**Authors:** Ikram Daidai

**Affiliations:** Department of Mathematics, Faculté des Sciences et Techniques, University Cadi Ayyad, B.P. 549, Marrakech, Morocco

**Keywords:** strong convexity of order *γ*, second-order approximation, $C^{1,1}$ functions, Newton’s method

## Abstract

It is well known that second-order information is a basic tool notably in optimality conditions and numerical algorithms. In this work, we present a generalization of optimality conditions to strongly convex functions of order *γ* with the help of first- and second-order approximations derived from (Optimization 40(3):229-246, 2011) and we study their characterization. Further, we give an example of such a function that arises quite naturally in nonlinear analysis and optimization. An extension of Newton’s method is also given and proved to solve Euler equation with second-order approximation data.

## Introduction

The concept of approximations of mappings was introduced by Thibault [2]. Sweetser [3] considered approximations by subsets of the space of continuous linear maps $L(X,Y)$, where *X* and *Y* are Banach spaces, and Ioffe [4] by the so-called fans. This approach was revised by Jourani and Thibault [5]. Another approach belongs to Allali and Amahroq [1]. Following the same ideas, Amahroq and Gadhi [6, 7] have established optimality conditions to some optimization problems under set-valued mapping constraints.

In this work, we explore the notion of strongly convex functions of order *γ*; see, for instance, [8–15] and references therein. Let *f* be a mapping from a Banach space *X* into $\mathbb{R}$, and let $C\subset X$ be a closed convex set. It is well known that the notion of strong convexity plays a central role. On the one hand, it ensures the existence and uniqueness of the optimal solution for the problem

$$(\mathcal{P})\quad \min_{x\in C} f(x). $$

On the other hand, if *f* is twice differentiable, then the strong convexity of *f* implies that its Hessian matrix is nonsingular, which is an important tool in numerical algorithms. Here we adopt the definition of a second-order approximation [1] to detect some equivalent properties of strongly convex functions of order *γ* and to characterize the latter. Furthermore, for a $C^{1,1}$ function *f* on a finite-dimensional setting, we show some simple facts. We also provide an extension of Newton’s method to solve an Euler equation with second-order approximation data.

The rest of the paper is written as follows. Section 2 contains basic definitions and preliminary results. Section 3 is devoted to mains results. In Section 4, we point out an extension of Newton’s method and prove its local convergence.

## Preliminaries

Let *X* and *Y* be two Banach spaces. We denote by $\mathcal{L}(X,Y)$ the set of all continuous linear mappings from *X* into *Y*, by $\mathcal{B}(X\times X,Y)$ the set of all continuous bilinear mappings from $X\times X$ into *Y*, and by $\mathbb{B}_{Y}$ the closed unit ball of *Y* centered at the origin.

Throughout this paper, $X^{*}$ and $Y^{*}$ denote the continuous duals of *X* and *Y*, respectively, and we write $\langle\cdot,\cdot\rangle$ for the canonical bilinear forms with respect to the dualities $\langle X^{*},X\rangle$ and $\langle Y^{*},Y\rangle$.

### Definition 1

[1]

Let *f* be a mapping from *X* into *Y*, $\bar{x}\in X$. A set of mappings $\mathcal{A}_{f}(\bar{x})\subset\mathcal{L}(X,Y)$ is said to be a first-order approximation of *f* at *x̄* if there exist $\delta >0$ and a function $r: X\to\mathbb{R}$ satisfying $\lim _{x\to \bar {x} }r(x)=0$ such that

1 $$ f(x)-f(\bar{x})\in\mathcal{A}_{f}(\bar{x}) (x-\bar{x})+ \Vert x-\bar{x} \Vert r(x)\mathbb{B}_{Y} $$

for all $x\in\bar{x} +\delta\mathbb{B}_{X}$.

It is easy to check that Definition 1 is equivalent to the following: for all $\varepsilon>0$, there exists $\delta>0$ such that

2 $$ f(x)-f(\bar{x})\in\mathcal{A}_{f}(\bar{x}) (x-\bar{x})+ \varepsilon \Vert x-\bar{x} \Vert \mathbb{B}_{Y} $$

for all $x\in\bar{x} +\delta\mathbb{B}_{X}$.

### Remark 1

If $\mathcal{A}_{f}(\bar{x})$ is a first-order approximation of *f* at *x̄*, then (2) means that for any $x\in\bar{x} +\delta\mathbb{B}_{X}$, there exist $A(x)\in\mathcal {A}_{f}(\bar{x})$ and $b\in\mathbb{B}_{Y}$ such that

$$f(x)-f(\bar{x})=A(x) (x-\bar{x})+\varepsilon \Vert x-\bar{x} \Vert b. $$

Hence, for any $x\in\mathbb{B}(\bar{x},\delta)$ and $A(x)\in \mathcal{A}_{f}(\bar{x})$,

3 $$ \bigl\Vert f(x)-f(\bar{x})-A(x) (x-\bar{x}) \bigr\Vert \leq \varepsilon \Vert x-\bar{x} \Vert . $$

If $\mathcal{A}_{f}(\bar{x})$ is norm-bounded (resp. compact), then it is called a bounded (resp. compact) first-order approximation. Recall that $\mathcal{A}_{f}(\bar{x})$ is a singleton if and only if *f* is Fréchet differentiable at *x̄*.

The following proposition proved by Allali and Amahroq [1] plays an important role in the sequel in a finite-dimensional setting.

### Proposition 1

[1]

*Let* $f: \mathbb{R}^{p} \to\mathbb{R}$ *be a locally Lipschitz function at* *x̄*. *Then the Clarke subdifferential of* *f* *at* *x̄*,

4 $$ \partial_{c}f (\bar{x}):=\operatorname{co} \bigl\{ \lim\nabla f(x_{n}): x_{n}\in \operatorname{dom} \nabla f\textit{ and }x_{n}\to\bar{x} \bigr\} , $$

*is a first*-*order approximation of* *f* *at* *x̄*.

In [6], it is also shown that when *f* is a continuous function, it admits as an approximation the symmetric subdifferential defined and studied in [16].

The next proposition shows that Proposition 1 holds also when *f* is a vector-valued function. Let us first recall the definition of the generalized Jacobian for a vector-valued function (see [17, 18] for more details) and the definition of upper semicontinuity.

### Definition 2

The generalized Jacobian of a function $g: \mathbb{R}^{p} \to\mathbb {R}^{q}$ at *x̄*, denoted $\partial_{c} g(\bar{x})$, is the convex hull of all matrices *M* of the form

$$M=\underset{n \to+\infty}{\lim} Jg(x_{n}), $$

where $x_{n}\to\bar{x}$, *g* is differentiable at $x_{n}$ for all *n*, and *Jg* denotes the $q\times p$ usual Jacobian matrix of partial derivatives.

### Definition 3

A set-valued mapping $F: \mathbb{R}^{p} \rightrightarrows\mathbb {R}^{q}$ is said to be upper semicontinuous at a point $\bar{x}\in \mathbb {R}^{p}$ if, for every $\varepsilon>0$, there exists $\delta>0$ such that

$$F(x)\subset F(\bar{x}) +\varepsilon\mathbb{B} $$

for every $x\in\mathbb{R}^{p}$ such that $\Vert x-\bar{x} \Vert <\delta$.

### Proposition 2

*Let* $g: \mathbb{R}^{p} \to\mathbb{R}^{q}$ *be a locally Lipschitz function at* *x̄*. *Then the generalized Jacobian* $\partial_{c} g(\bar {x})$ *of* *g* *at* *x̄* *is a first*-*order approximation of* *g* *at* *x̄*.

### Proof

Since the set-valued mapping $\partial_{c} g(\cdot)$ is upper semicontinuous, for all $\varepsilon>0$, there exists $r_{0}>0$ such that

$$\partial_{c} g(x)\subset\partial_{c} g(\bar{x})+ \varepsilon\mathbb{B}_{\mathcal{L}(\mathbb{R}^{p},\mathbb{R}^{q})}\quad \mbox{for all } x\in\bar{x} +r_{0} \mathbb{B}_{\mathbb{R}^{p}}. $$

We may assume that *g* is Lipschitzian in $\bar{x} +r_{0}\mathbb {B}_{\mathbb{R}^{p}}$. Let $x\in\bar{x} +r_{0}\mathbb{B}_{\mathbb{R}^{p}}$. We apply [17], Prop. 2.6.5, to derive that there exits $c\in\mathopen{]}x,\bar{x}[$ such that

$$g(x)-g(\bar{x}) \in\partial_{c} g(c) (x-\bar{x})\subset \partial_{c} g(\bar{x}) (x-\bar{x})+ \varepsilon\mathbb{B}_{\mathcal {L}(\mathbb {R}^{p},\mathbb{R}^{q})}(x- \bar{x}). $$

Since

$$\mathbb{B}_{\mathcal{L}(\mathbb{R}^{p},\mathbb{R}^{q})}(x-\bar {x})\subset \Vert x-\bar{x} \Vert \mathbb{B}_{\mathbb{R}^{q}}, $$

we have

$$g(x)-g(\bar{x}) \in\partial_{c} g(\bar{x}) (x-\bar{x})+ \varepsilon \Vert x-\bar{x} \Vert \mathbb{B}_{\mathbb{R}^{q}}, $$

which means that $\partial_{c} g(\bar{x})$ is a first-order approximation of *g* at *x̄*. □

Recall that a mapping $f: X \to Y$ is said to be $C^{1,1}$ at *x̄* if it is Fréchet differentiable in neighborhood of *x̄* and if its Fréchet derivative $\nabla f(\cdot)$ is Lipschitz at *x̄*.

Let $\bar{x}\in\mathbb{R}^{p}$, and let $f: \mathbb{R}^{p} \rightarrow \mathbb{R}$ be a $C^{1,1}$ function at *x̄*. The generalized Hessian matrix of *f* at *x̄* was introduced and studied by Hiriart-Urruty et al. [19] is the compact nonempty convex set

5 $$ \partial^{2}_{H} f(\bar{x}):=\operatorname{co} \bigl\{ \lim \nabla^{2} f(x_{n}): (x_{n}) \in \operatorname{dom} \nabla^{2} f \textit{ and } x_{n} \to\bar{x} \bigr\} , $$

where $\operatorname{dom} \nabla^{2} f$ is the effective domain of $\nabla^{2} f(\cdot)$.

### Corollary 1

*Let* $\bar{x}\in\mathbb{R}^{p}$, *and* $f: \mathbb{R}^{p} \rightarrow \mathbb{R}$ *be a* $C^{1,1}$ *function at* *x̄*. *Then*, ∇*f* *admits* $\partial^{2}_{H} f(\bar{x})$ *as a first*-*order approximation at* *x̄*.

### Definition 4

[1]

We say that $f: X \rightarrow Y$ admits a second-order approximation at *x̄* if there exit two sets $\mathcal{A}_{f} (\bar{x})\subset \mathcal{L}(X,Y)$ and $\mathcal{B}_{f} (\bar{x})\subset\mathcal {B}(X\times X,Y)$ such that

(i) $\mathcal{A}_{f} (\bar{x})$ is a first-order approximation of *f* at *x̄*;
(ii) For all $\varepsilon>0$, there exists $\delta>0$ such that
  $$f(x)-f(\bar{x})\in\mathcal{A}_{f} (\bar{x}) (x-\bar{x})+ \mathcal{B}_{f} (\bar{x}) (x-\bar{x}) (x-\bar{x})+\varepsilon \Vert x- \bar{x} \Vert ^{2}\mathbb{B}_{Y} $$
  for all $x\in\bar{x}+\delta\mathbb{B}_{X}$.

In this case the pair $(\mathcal{A}_{f} (\bar{x}),\mathcal{B}_{f} (\bar {x}))$ is called a second-order approximation of *f* at *x̄*. It is called a compact second-order approximation if $\mathcal{A}_{f} (\bar {x})$ and $\mathcal{B}_{f} (\bar{x})$ are compacts.

Every $C^{2}$ mapping $f: X \to Y$ at *x̄* admits $(\nabla f(\bar {x}), \nabla^{2} f(\bar{x}))$ as a second-order approximation, where $\nabla f(\bar{x})$ and $\nabla^{2} f(\bar{x})$ are, respectively, the first- and second-order Fréchet derivatives of *f* at *x̄*.

### Proposition 3

[1]

*Let* $f: \mathbb{R}^{p} \rightarrow\mathbb{R}$ *be a* $C^{1,1}$ *function at* *x̄*. *Then* *f* *admits* $(\nabla f(\bar{x}),\frac {1}{2}\partial ^{2}_{H} f(\bar{x}))$ *as a second*-*order approximation at* *x̄*.

### Proposition 4

*Let* $f: X\to Y$ *be a Fréchet*-*differentiable mapping*. *If* $(\nabla f(\bar{x}),\mathcal{B}_{f}(\bar{x}))$ *is a bounded second*-*order approximation of* *f* *at* *x̄*. *Then* $\nabla f(\cdot)$ *is stable at* *x̄*, *that is*, *there exist* $c, r>0$ *such that*

6 $$ \bigl\Vert \nabla f(x)-\nabla f(\bar{x}) \bigr\Vert \leq c \Vert x-\bar{x} \Vert $$

*for all* $x\in\bar{x} +r\mathbb{B}_{X}$.

To derive some results for *γ*-strong convex functions, the following notions are needed.

### Definition 5

[8]

Let $\gamma>0$. We say that a map $f: X \to\mathbb{R}\cup\{ +\infty\}$ is *γ*-strongly convex if there exist $c\geq0$ and $g: [0,1]\to\mathbb{R}^{+}$ satisfying

7 $$ g(0)=g(1)=0 \quad\mbox{and}\quad \underset{\theta\to0}{\lim} \frac {g(\theta )}{\theta}=1 $$

and such that

8 $$ f \bigl(\theta x+(1-\theta)y \bigr)\leq\theta f(x)+(1- \theta)f(y)-c g(\theta) \Vert x-y \Vert ^{\gamma} $$

for all $\theta\in[0,1]$ and $x, y\in X$.

Of course, when $c=0$, *f* is called a convex function. Otherwise, *f* is said *γ*-strongly convex. This class has been introduced by Polyak [11] when $\gamma=2$ and $g(\theta)=\theta(1-\theta)$ and studied by many authors. Recently, a characterization of *γ*-strongly convex functions has been shown in [8]. For example, if *f* is $C^{1}$ and $\gamma\geq1$, then (8) is equivalent to

9 $$ \bigl\langle \nabla f(x),y-x \bigr\rangle \leq f(y)-f(x)- \frac{c}{\gamma} \Vert y-x \Vert ^{\gamma},\quad \forall x, y\in X. $$

Let $f: X \to\mathbb{R}\cup\{+\infty\}$ and $\bar{x} \in \operatorname{dom} f:=\{x\in X, f(x)<+\infty\}$ (the effective domain of *f*). The Fenchel-subdifferential of *f* at *x̄* is the set

10 $$ \partial_{\mathrm{Fen}} f(\bar{x})= \bigl\{ x^{*}\in X^{*}: \bigl\langle x^{*},y-\bar{x} \bigr\rangle \leq f(y)-f(\bar{x}), \forall y\in X \bigr\} . $$

Let $\gamma>0$ and $c>0$. The $(\gamma, c)$-subdifferential of *f* at *x̄* is the set

11 $$ \partial_{(\gamma, c)} f(\bar{x})= \bigl\{ x^{*}\in X^{*}: \bigl\langle x^{*},y-\bar{x} \bigr\rangle \leq f(y)-f(\bar{x}) - c \Vert \bar {x}-y \Vert ^{\gamma }, \forall y\in X \bigr\} . $$

For more details on $(\gamma, c)$-subdifferential, see [8]. Note that if $x\notin \operatorname{dom} f$, then $\partial_{(\gamma,c)} f(\bar {x})=\partial_{\mathrm{Fen}} f(\bar{x})=\emptyset$. Clearly, we have $\partial_{(\gamma,c)}f(\bar{x})\subset\partial_{\mathrm{Fen}} f(\bar{x})$. Note that the Fenchel-subdifferential defined by (10) coincides with the Clarke subdifferential of *f* at *x̄* if the function *f* is convex. We also need to recall the following definitions.

### Definition 6

[20]

We say that a map $f: X \to\mathbb{R}\cup\{+\infty\}$ is 2-paraconvex if there exists $c>0$ such that

12 $$ f \bigl(\theta x+(1-\theta)y \bigr)\leq\theta f(x)+(1- \theta)f(y)+c \min(\theta,1-\theta) \Vert x-y \Vert ^{2} $$

for all $\theta\in[0,1]$ and $x, y\in X$.

It has been proved in [20] that if *f* is a $C^{1}$ mapping, then (12) is equivalent to

13 $$ \bigl\langle \nabla f(x),y-x \bigr\rangle \leq f(y)-f(x)+c \Vert y-x \Vert ^{2}, \quad\forall x, y\in X. $$

## Main results

In this section, we obtain the main results of the paper related to strongly convex functions of order *γ* defined by (7)-(8). We begin by showing some interesting facts of functions that admit a first-order approximation.

For any subset *A* of $X^{*}$, we define the support function of *A* as

14 $$ s(A,x)=\sup \bigl\{ \bigl\langle x^{*},x \bigr\rangle , x^{*}\in A \bigr\} . $$

It is well known that, for any convex function *f*: $X\rightarrow \mathbb{R}\cup\{+\infty\}$, the ‘right-hand’ directional derivative at *x* in dom*f* (the domain of *f* ) exists and, for each $h\in X$, is

$$d^{+}f(x) (h)=\underset{t \rightarrow0^{+}}{\lim}\frac{f(x+th)-f(x)}{t}. $$

### Theorem 1

*Let* $\bar{x}\in X$. *If* $f:X\to\mathbb{R}\cup\{+\infty\}$ *is convex and continuous at* *x̄* *and if* $\mathcal{A}_{f}(\bar{x})\subset X^{*}$ *is a convex* $w(X^{*},X)$-*closed approximation of* *f* *at* *x̄*, *then*

$$\partial_{(\gamma,c)}f(\bar{x})\subset\mathcal{A}_{f}(\bar{x}). $$

### Proof

By the definition of $\mathcal{A}_{f} (\bar{x})$, there exist $\delta >0$ and $r:X \to\mathbb{R}$ with $\lim_{x\to\bar{x}} r(x)=0$ such that, for all $x\in\bar{x}+\delta\mathbb{B}_{X}$, $t\in ]0,\delta[$, and $h\in X$, there exist $A\in\mathcal{A}_{f} (\bar{x})$ and $b\in[-1,1]$ satisfying

$$\frac{f(\bar{x}+th)-f(\bar{x})}{t} - \Vert h \Vert r(\bar {x}+th)b=\langle A,h\rangle\leq s \bigl(\mathcal{A}_{f} (\bar{x});h \bigr). $$

By letting $t\to0^{+}$ the directional derivative of *f* at *x̄* satisfies

15 $$ d^{+}f(\bar{x}) (h)\leq s \bigl(\mathcal{A}_{f} (\bar{x});h \bigr),\quad \forall h\in X. $$

Using [21], Prop. 2.24, we get

$$s \bigl(\partial_{\mathrm{Fen}} f (\bar{x});h \bigr)\leq s \bigl( \mathcal{A}_{f} (\bar{x});h \bigr). $$

Since $\partial_{(\gamma,c)}f(\bar{x})\subset\partial_{\mathrm{Fen}} f(\bar {x})$, we deduce that

$$s \bigl(\partial_{(\gamma,c)}f(\bar{x});h \bigr)\leq s \bigl( \mathcal{A}_{f} (\bar{x});h \bigr). $$

Hence we conclude that $\partial_{(\gamma,c)}f(\bar{x})\subset \mathcal {A}_{f} (\bar{x})$. □

### Proposition 5

*Let* $f: X \to\mathbb{R}\cup\{+\infty\}$ *be a* *γ*-*strongly convex function*. *Assume that* $\mathcal{A}_{f}(\bar{x})$ *is a compact approximation at* *x̄*. *Then* $\mathcal{A}_{f}(\bar{x})\cap \partial _{(\gamma,c)}f(\bar{x})\neq \emptyset$.

### Proof

Let $d\in X$ be fixed and define $x_{n}:=\bar{x}+\frac{1}{n}d$. Using Definition 1, we get, for *n* large enough, $A_{n}\in\mathcal {A}_{f}(\bar{x})$ and $b_{n}\in[-1,1]$ such that

$$\frac{1}{n}\langle A_{n},d\rangle=f \biggl(\bar{x}+ \frac{1}{n}d \biggr)-f(\bar{x})-\frac {1}{n} \Vert d \Vert r(x_{n})b_{n}. $$

By *γ*-strong convexity we obtain

$$\frac{1}{n}\langle A_{n},d\rangle\leq\frac{1}{n} \bigl( f( \bar{x}+d)-f(\bar{x}) \bigr)-c g \biggl(\frac{1}{n} \biggr) \Vert d \Vert ^{\gamma}- \frac {1}{n} \Vert d \Vert r(x_{n})b_{n}. $$

By the compactness of $\mathcal{A}_{f}(\bar{x})$, extracting a subsequence if necessary, we may assume that there exists $A\in \mathcal {A}_{f}(\bar{x})$ such that $\langle A_{n},d\rangle \to\langle A,d\rangle $; and hence we obtain

16 $$ \langle A,d\rangle \leq f(\bar{x}+d)-f(\bar{x}) -c \Vert d \Vert ^{\gamma}. $$

Assume that $A\in\mathcal{A}_{f}(\bar{x})\cap\partial_{(\gamma ,c)}f(\bar {x})$. By the separation theorem there exists $h\in X$ with $\Vert h \Vert =1$ such that

$$\min_{A\in\mathcal{A}_{f} (\bar{x}) }\langle A,h\rangle > \sup_{x^{*}\in\partial _{(\gamma,c)} f(\bar{x})} \bigl\langle x^{*},h\bigr\rangle . $$

Let $t >0$ sufficiently small, so that

$$\min_{A\in\mathcal{A}_{f} (\bar{x}) }\langle A,h\rangle >\frac {f(\bar{x}+th)-f(\bar{x})}{t}, $$

in contradiction with relation (16) by taking $d=th$. □

Following a result by Rademacher, which states that a locally Lipschitzian function between finite-dimensional spaces is differentiable (Lebesgue) almost everywhere, we can prove the following result.

### Proposition 6

*Let* $\gamma\geq1$, $\bar{x}\in\mathbb{R}^{p}$, *and let* $f: \mathbb {R}^{p} \to\mathbb{R}$ *be continuous at* *x̄*. *Assume that* *f* *is a* *γ*-*strongly convex function*. *Then* $\partial_{c} f (\bar{x})= \partial_{(\gamma,c)}f(\bar{x})$.

### Proof

Obviously, we have $\partial_{(\gamma,c)}f(\bar{x})\subset\partial_{c} f (\bar{x})$. Now let $A\in\partial_{c} f (\bar{x})$. For all *n*, there exists $x_{n}\in \operatorname{dom} \nabla f$ such that $x_{n}\to\bar{x}$ and $\nabla f(x_{n})\to A$. Since *f* is *γ*-strongly convex and Fréchet differentiable at $x_{n}$ for all $n\in\mathbb{N}$, it follows by (9) that

$$\bigl\langle \nabla f(x_{n}),y-x_{n}\bigr\rangle \leq f(y)-f(x_{n})-c \Vert y-x_{n} \Vert ^{\gamma}, \quad\forall y\in \mathbb{R}^{p}, \forall n\in\mathbb{N}. $$

Letting $n\to+\infty$, we get

$$\langle A,y-\bar{x}\rangle \leq f(y)-f(\bar{x})-c \Vert y-\bar {x} \Vert ^{\gamma},\quad \forall y\in\mathbb{R}^{p}, $$

which means that $\partial_{c} f (\bar{x}) \subset\partial_{(\gamma ,c)}f(\bar{x})$. □

### Corollary 2

*Let* $\gamma\geq1$, $\bar{x}\in\mathbb{R}^{p}$, *and let* $f: \mathbb {R}^{p} \to\mathbb{R}$ *be continuous at* *x̄*. *Assume that* *f* *is a* *γ*-*strongly convex function*. *Then*, *for all* $\varepsilon>0$, *there exists* $r>0$ *such that*

17 $$ f(x)-f(\bar{x})\in\partial_{(\gamma,c)} f(\bar{x}) (x-\bar {x})+\varepsilon \Vert x-\bar{x} \Vert \mathbb{B}_{\mathbb{R}} $$

*for all* $x\in\bar{x}+r\mathbb{B}_{\mathbb{R}^{p}}$, *which means that* $\partial_{(\gamma,c)} f(\bar{x})$ *is a first*-*order approximation of* *f* *at* *x̄*.

### Proof

It is clear that $\partial_{c} f (\bar{x})$ is a first-order approximation of at *x̄*. We end the proof by Propositions 1 and 6. □

The converse of Proposition 5 holds if (16) is valid for any $A\in\mathcal{A}_{f}(x)$ and $x\in X$.

### Proposition 7

*Let* $\gamma\geq1$ *and* $f:X\to\mathbb{R}\cup\{+\infty\}$. *Assume that*, *for each* $x\in X$, *f* *admits a first*-*order approximation* $\mathcal{A}_{f}(x)$ *such that* $\mathcal{A}_{f}(x)\subset\partial _{(\gamma ,c)} f(x)$. *Then* *f* *is* *γ*-*strongly convex*.

### Proof

Define $x_{\theta}:=\theta u+(1-\theta)v$ for $\theta\in[0,1]$ and $u, v\in X$. Let us take $A\in\mathcal{A}_{f} (x_{\theta})$. Then

$$\langle A,u-x_{\theta}\rangle \leq f(u)-f(x_{\theta})-c \Vert u-x_{\theta } \Vert ^{\gamma}. $$

Multiplying this inequality by *θ*, we obtain

$$\bigl(\mathrm{a}' \bigr)\quad \theta(1-\theta)\langle A,u-v\rangle \leq\theta f(u)-\theta f(x_{\theta})-c(1-\theta)^{\gamma} \theta \Vert u-v \Vert ^{\gamma}. $$

In a similar way, since

$$\langle A,v-x_{\theta}\rangle \leq f(v)-f(x_{\theta})-c \Vert v-x_{\theta } \Vert ^{\gamma}, $$

we get

$$\bigl(\mathrm{a}'' \bigr)\quad {-}\theta(1-\theta)\langle A,u-v\rangle \leq(1- \theta) f(v)- (1-\theta) f(x_{\theta})-c(1-\theta) \theta^{\gamma} \Vert u-v \Vert ^{\gamma}. $$

We deduce by addition of $(\mathrm{a}')$ and $(\mathrm{a}'')$ that

$$f(x_{\theta})\leq\theta f(u)+(1-\theta) f(v)-cg(\theta) \Vert u-v \Vert ^{\gamma} \quad\mbox{for all } u, v\in X, $$

where $g(\theta)=(1-\theta) \theta^{\gamma} +(1-\theta)^{\gamma} \theta $, so that *f* is *γ*-strongly convex. □

The next results are devoted to presenting some useful properties of the generalized Hessian matrix for a $C^{1,1}$ function in the finite-dimensional setting and a characterization of *γ*-strongly convex functions with the help of a second-order approximation.

### Proposition 8

*Let* $\bar{x}\in X$, *and let* $f: X \rightarrow\mathbb{R}\cup\{ +\infty\}$ *be convex and Fréchet differentiable at* *x̄*. *Suppose that* *f* *admits* $(\nabla f(\bar{x}),\mathcal{B}_{f}(\bar{x}))$ *as a second*-*order approximation at* *x̄* *and that* $\mathcal {B}_{f}(\bar {x})$ *is compact*. *Then there exists* $B\in\mathcal{B}_{f}(\bar{x})$ *such that*

18 $$ \sup_{B\in\mathcal{B}_{f}(\bar{x})}\langle Bd,d\rangle \geq0,\quad \forall d \in X. $$

*If* *f* *is* 2-*strongly convex*, *then we obtain*

19 $$ \sup_{B\in\mathcal{B}_{f}(\bar{x})} \langle Bd,d\rangle \geq c \Vert d \Vert ^{2},\quad \forall d\in X, $$

*for some* $c>0$.

### Proof

We prove only the case where *f* is convex. In a similar way, we can prove the other case. Let $d\in X$ and $\varepsilon>0$ be fixed. We get for *n* large enough $B_{n}\in\mathcal{B}_{f}(\bar{x})$ and $b_{n}\in [-1,1]$ such that

$$f \biggl(\bar{x}+\frac{1}{n}d \biggr)-f(\bar{x})=\frac {1}{n}\bigl\langle \nabla f(\bar{x}),d\bigr\rangle +\frac{1}{n^{2}}\langle B_{n} d,d\rangle +\varepsilon \frac{1}{n^{2}} \Vert d \Vert ^{2}b_{n}. $$

Since *f* is convex, we obtain

$$\langle B_{n} d,d\rangle +\varepsilon \Vert d \Vert ^{2}b_{n} \geq0. $$

By the compactness of $\mathcal{B}_{f}(\bar{x})$, extracting a subsequence if necessary, we may assume that there exits $B\in \mathcal{B}_{f}(\bar{x})$ such that $B_{n}$ converges to *B*; therefore

$$\langle Bd,d\rangle \geq0, $$

and hence

$$\sup_{B\in\mathcal{B}_{f}(\bar{x})}\langle Bd,d\rangle \geq0, \quad \forall d\in X. $$

 □

When *X* is a finite-dimensional space, we get the following essential result.

### Proposition 9

*Let* $f: \mathbb{R}^{p} \rightarrow\mathbb{R}$ *be a* $C^{1,1}$ *function at* *x̄*. *Assume that* *f* *is* *γ*-*strongly convex*. *Then*, *for any* $B\in\partial^{2}_{H} f(\bar{x})$, *we have the following inequality*:

20 $$ \langle Bd,d\rangle \geq c \Vert d \Vert ^{\gamma},\quad \forall d\in \mathbb{R}^{p}, $$

*for some* $c>0$.

### Proof

It is clear that $(\nabla f(\bar{x}),\frac{1}{2}\partial^{2}_{H} f(\bar {x}))$ is a second-order approximation of *f* at *x̄*. Now let $B\in\partial^{2}_{H} f(\bar{x})$, so that there exists a sequence $(x_{n})\in \operatorname{dom} \nabla^{2} f$ such that $x_{n}\to\bar{x}$ and $\nabla^{2} f(x_{n})\to B$. Since *f* is *γ*-strongly convex, there exists $c>0$ such that

$$\bigl\langle \nabla^{2} f(x_{n}) d,d\bigr\rangle \geq c \Vert d \Vert ^{\gamma}, \quad\forall d\in\mathbb{R}^{p}, \forall n \in\mathbb{N}. $$

Letting $n\to+\infty$, we have

$$\langle Bd,d\rangle \geq c \Vert d \Vert ^{\gamma},\quad \forall d\in \mathbb{R}^{p}. $$

 □

The preceding result shows that *γ*-strongly convex functions enjoy a very desirable property for generalized Hessian matrices. In fact, in this case, any matrix $B\in\partial^{2}_{H} f(\bar{x})$ is invertible. The next result proves the converse of Proposition 9. Let us first recall the following characterization of l.s.c. *γ*-strongly convex functions.

### Theorem 2

Amahroq et al. [8]

*Let* *f*: $X\rightarrow \mathbb{R}\cup\{+\infty\}$ *be a proper and l*.*s*.*c*. *function*. *Then f is* *γ*-*strongly convex iff* $\partial_{c} f$ *is* *γ*-*strongly monotone*, *that is*, *there exists a positive real number c such that*, *for all* $x, y\in X$, $x^{*}\in\partial_{c} f(x)$, *and* $y^{*} \in\partial_{c} f(y)$, *we have*

$$\bigl\langle x^{*}-y^{*},x-y\bigr\rangle \geq c \Vert x-y \Vert ^{\gamma}. $$

We are now in position to state our main second result.

### Theorem 3

*Let* $f: \mathbb{R}^{p} \rightarrow\mathbb{R}$ *be a* $C^{1,1}$ *function*. *Assume that* $\partial^{2}_{H} f(\cdot)$ *satisfies relation* (20) *at any* $x\in\mathbb{R}^{p}$. *Then* *f* *is* *γ*-*strongly convex*.

### Proof

Let $t\in[0,1]$ and $u, v\in\mathbb{R}^{p}$. Define $\varphi:\mathbb {R}\to\mathbb{R}$ as

$$\varphi(t):=f \bigl(u+t(v-u) \bigr), $$

so that $\varphi'(t):=\langle \nabla f(u+t(v-u)),v-u\rangle $. By the Lebourg mean value theorem [22] there exists $t_{0}\in\mathopen{]}0,1[$ such that

$$\varphi'(1)-\varphi'(0)\in\partial_{c} \varphi'(t_{0}). $$

By using calculus rules it follows that

$$\varphi'(1)-\varphi'(0)\in\partial_{c} \varphi'(t_{0})\subset\partial^{2}_{H} f \bigl(u+t_{0}(v-u) \bigr) (v-u) (v-u). $$

Hence, there exists $B_{t_{0}} \in\partial^{2}_{H} f(u+t_{0}(v-u))$ such that $\langle \nabla f(v)-\nabla f(u),v-u\rangle =\langle B_{t_{0}} (v-u),v-u\rangle $. The result follows from Theorem 2. □

Hiriart-Urruty et al. [19] have presented many examples of $C^{1,1}$ functions. The next proposition shows another example of a $C^{1,1}$ function.

### Theorem 4

*Let* $f: H \rightarrow\mathbb{R}$ *be continuous on a Hilbert space H*. *Suppose that* *f* *is convex* (*or* 2-*strongly convex*) *and that* −*f* *is* 2-*paraconvex*. *Then* *f* *is Fréchet differentiable on H*, *and for some* $c>0$, *we have that*

21 $$ \bigl\Vert \nabla f(x)-\nabla f(y) \bigr\Vert \leq c \Vert x-y \Vert \quad\textit{for all }x, y\in H. $$

### Proof

Let $x_{0}\in X$. Clearly, *f* is locally Lipschitzian at $x_{0}$. Now let $x_{1}^{*}$ and $x_{2}^{*}$ be arbitrary elements of $\partial_{c} f(x_{0})$ and $\partial_{c} (-f)(x_{0})$, respectively. By [20], Thm. 3.4, there exists $c>0$ such that $\partial_{c} (-f)(x_{0})=\partial^{(2,c)} (-f)(x_{0})$, and for any $y\in H$ and positive real *θ*, we have

$$(\mathrm{a})\quad \theta\bigl\langle x_{2}^{*},y\bigr\rangle \leq-f(x_{0}+ \theta y)+f(x_{0})+c \theta^{2} \Vert y \Vert ^{2} $$

and

$$\bigl(\mathrm{a}' \bigr)\quad \theta\bigl\langle x_{1}^{*},y\bigr\rangle \leq f(x_{0}+\theta y)-f(x_{0}). $$

Adding (a) and (a′), we get

$$\theta\bigl\langle x_{1}^{*}+x_{2}^{*},y\bigr\rangle \leq c \theta^{2} \Vert y \Vert ^{2}, $$

and hence

$$\bigl\langle x_{1}^{*}+x_{2}^{*},y\bigr\rangle \leq c \theta \Vert y \Vert ^{2}. $$

Letting $\theta\to0$, we have $\langle x_{1}^{*}+x_{2}^{*},y\rangle \leq 0$, so that $x_{1}^{*}=-x_{2}^{*}$. Since $x_{1}^{*}$ and $x_{2}^{*}$ are arbitrary in $\partial_{c} f(x_{0})$ and $\partial_{c} (-f)(x_{0})$, it follows that $\partial_{c} f(x_{0})$ is single-valued. Put $\partial_{c} f(x_{0})=\{p(x_{0})\}$. Since (a) and (a′) hold for any $\theta> 0 $ and $y\in H$, we deduce that, for $\theta=1$,

$$\bigl\langle p(x_{0}),y\bigr\rangle \leq f(x_{0}+y)-f(x_{0}) $$

and

$$f(x_{0}+y)-f(x_{0})-\bigl\langle p(x_{0}),y \bigr\rangle \leq c \Vert y \Vert ^{2}. $$

Hence, for all $y\neq0$, we obtain

22 $$ \frac{ | f(x_{0}+ y)-f(x_{0})-\langle p(x_{0}),y\rangle |}{ \Vert y \Vert } \leq c \Vert y \Vert . $$

Letting $\Vert y \Vert \to0$ in (22), we conclude that *f* is Fréchet differentiable at $x_{0}$. Now since −*f* is 2-paraconvex and *f* is Fréchet differentiable, we may prove that there exists $c>0$ such that

23 $$ -\bigl\langle \nabla f(x),y-x\bigr\rangle \leq-f(y)+f(x)+c \Vert x-y \Vert ^{2} \quad\mbox{for all } x, y\in H. $$

For every $z\in H$, we have that

$$-f(z)\geq-f(x)+\bigl\langle \nabla f(x),x\bigr\rangle -\bigl\langle \nabla f(x),z\bigr\rangle -c \Vert x-z \Vert ^{2}. $$

Thus

$$-f(z)\geq f^{*} \bigl(\nabla f(x) \bigr)-\bigl\langle \nabla f(x),z\bigr\rangle -c \Vert x-z \Vert ^{2}, $$

so that

$$\begin{aligned} &f^{*} \bigl(\nabla f(y) \bigr)\geq\bigl\langle \nabla f(y),z\bigr\rangle -f(z), \\ &f^{*} \bigl(\nabla f(y) \bigr)\geq\bigl\langle \nabla f(y),z\bigr\rangle +f^{*} \bigl(\nabla f(x) \bigr)-\bigl\langle \nabla f(x),z\bigr\rangle -c \Vert x-z \Vert ^{2}, \end{aligned}$$

and hence

$$\begin{aligned} &f^{*} \bigl(\nabla f(y) \bigr)- f^{*} \bigl(\nabla f(x) \bigr)-\bigl\langle \nabla f(y)-\nabla f(x),x\bigr\rangle \\ &\quad\geq\bigl\langle \nabla f(y)-\nabla f(x),z-x\bigr\rangle -c \Vert x-z \Vert ^{2} \\ &\quad\geq\underset{z\in H}{\sup} \bigl\{ \bigl\langle \nabla f(y)-\nabla f(x),z-x \bigr\rangle -c \Vert x-z \Vert ^{2} \bigr\} . \end{aligned}$$

This means that, for all $x, y \in H$,

$$f^{*} \bigl(\nabla f(y) \bigr)- f^{*} \bigl(\nabla f(x) \bigr)-\bigl\langle \nabla f(y)-\nabla f(x),x\bigr\rangle \geq\frac{1}{2c} \bigl\Vert \nabla f(y)- \nabla f(x) \bigr\Vert ^{2}. $$

Changing the roles of *x* and *y*, we obtain

$$f^{*} \bigl(\nabla f(x) \bigr)- f^{*} \bigl(\nabla f(y) \bigr)-\bigl\langle \nabla f(x)-\nabla f(y),y\bigr\rangle \geq\frac{1}{2c} \bigl\Vert \nabla f(x)- \nabla f(y) \bigr\Vert ^{2}. $$

So by addition we get

24 $$ \bigl\langle \nabla f(x)-\nabla f(y),x-y\bigr\rangle \geq \frac {1}{c} \bigl\Vert \nabla f(x)-\nabla f(y) \bigr\Vert ^{2}. $$

Consequently, by the Cauchy-Schwarz inequality we obtain

$$\bigl\Vert \nabla f(x)-\nabla f(y) \bigr\Vert \leq c \Vert x-y \Vert \quad \mbox{for all }x, y\in H. $$

 □

## Newton’s method

The aim of this section is to solve the Euler equation

25 $$ \nabla f(x)=0 $$

by Newton’s method. The classic assumption is that $f: \mathbb{R}^{p} \rightarrow\mathbb{R}$ a $C^{2}$ mapping and the Hessian matrix $\nabla ^{2} f(x)$ of *f* at *x* is nonsingular. Here we prove the convergence of a natural extension of Newton’s method to solve (25) assuming that $\nabla f(\cdot)$ admits $\beta_{f}(\cdot)$ as a first-order approximation. Clearly, if $f: \mathbb{R}^{p} \rightarrow\mathbb{R}$ is a $C^{1,1}$ mapping, then using Corollary 1, we obtain that $\nabla f(\cdot)$ admits $\partial_{H}^{2} f(\cdot)$ as a first-order approximation.

This algorithm has been proposed by Cominetti et al. [23] with $C^{1,1}$ data. Only some ideas were given, but it remains as an open question to state results on rate of convergence and local convergence of that algorithm. In the sequel, $f: \mathbb{R}^{p} \rightarrow\mathbb {R}$ is a Fréchet-differentiable mapping such that its Fréchet derivative admits a first-order approximation, and *x̄* is a solution of (25). 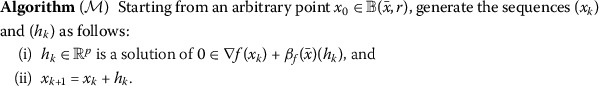


### Theorem 5

*Let* $f: \mathbb{R}^{p} \rightarrow\mathbb{R}$ *be a Fréchet*-*differentiable function*, *and* *x̄* *be a solution of* (25). *Let* $\varepsilon, r, K >0$ *be such that* $\nabla f(\cdot)$ *admits* $\beta_{f}(\bar{x})$ *as a first*-*order approximation at* *x̄* *such that*, *for each* $x\in\mathbb{B}_{\mathbb{R}^{p}} (\bar{x},r)$, *there exists an invertible element* $B(x) \in\mathcal{B}_{f}(x)$ *satisfying* $\Vert B(x)^{-1} \Vert \leq K$ *and* $\xi:= \varepsilon K<1$. *Then the sequence* $(x_{k})$ *generated by Algorithm* $(\mathcal {M})$ *is well defined for every* $x_{0} \in\mathbb{B}_{\mathbb{R}^{p}}(\bar {x},r)$ *and converges linearly to* *x̄* *with rate* *ξ*.

### Proof

Since $\nabla f(\bar{x})=0$, we have

$$x_{k+1}-\bar{x} =B(x_{k})^{-1} \bigl( \nabla f( \bar{x})-\nabla f(x_{k})+B(x_{k}) (x_{k} -\bar{x}) \bigr). $$

We inductively obtain that

$$\Vert x_{k+1}-\bar{x} \Vert \leq K \bigl\Vert \nabla f (\bar {x})- \nabla f(x_{k})+B(x_{k}) (x_{k}-\bar{x}) \bigr\Vert . $$

Thus

$$\Vert x_{k+1}-\bar{x} \Vert \leq\xi \Vert x_{k}- \bar{x} \Vert , $$

which means that $x_{k+1} \in\mathbb{B}_{\mathbb{R}^{p}}(\bar{x},r)$, and we have $\Vert x_{k+1}-\bar{x} \Vert \leq\xi^{k} \Vert x_{0}-\bar{x} \Vert $. Therefore the whole sequence $(x_{k})$ is well defined and converges to *x̄*. □

Now let us consider the following algorithm under less assumptions. 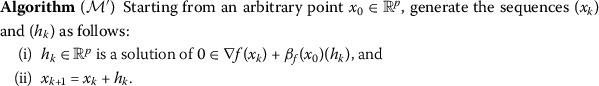


### Theorem 6

*Let* *U* *be an open set of* $\mathbb{R}^{p}$, $x_{0}\in U$, *and* $f: \mathbb {R}^{p} \rightarrow\mathbb{R}$ *be a Fréchet*-*differentiable function on* *U*. *Let* $\varepsilon, r, K >0$ *be such that* $\nabla f(\cdot)$ *admits* $\beta_{f}(x_{0})$ *as a strict first*-*order approximation at* $x_{0}$ *such that*, *for each* $x\in\mathbb{B}_{\mathbb{R}^{p}} (x_{0},r)$, *there exists a right inverse of* $B(x)\in\beta_{f}(x_{0})$, *denoted by* $\tilde {B}(x)$, *satisfying* $\Vert \tilde{B}(x)(\cdot) \Vert \leq K \Vert \cdot \Vert $ *and* $\xi:= \varepsilon K<1$.

*If* $\Vert \nabla f(x_{0}) \Vert \leq K^{-1}(1-\xi)r $ *and* ∇*f* *is continuous*, *then the sequence* $(x_{k})$ *generated by Algorithm* $(\mathcal {M}')$ *is well defined and converges to a solution* *x̄* *of* (25). *Moreover*, *we have* $\Vert x_{k}-\bar {x} \Vert \leq r\xi^{k}$ *for all* $k\in\mathbb{N}$ *and* $\Vert \bar {x}-x_{0} \Vert \leq \Vert \nabla f(x_{0}) \Vert K(1-\xi)^{-1}< r$.

### Proof

We prove by induction that $x_{k}\in x_{0} +r \mathbb{B}_{ \mathbb{R}^{p}}$, $\Vert x_{k+1}-x_{k} \Vert \leq K \xi^{k} \Vert \nabla f(x_{0}) \Vert $, and $\Vert \nabla f(x_{k}) \Vert \leq\xi ^{k} \Vert \nabla f(x_{0}) \Vert $ for all $k\in\mathbb{N}$. For $k=0$, these relations are obvious. Assuming that they are valid for $k< n$, we get

$$\begin{aligned} \Vert x_{n} -x_{0} \Vert &\leq\underset{k=0}{ \overset{n-1}{\sum}} \Vert x_{k+1}-x_{k} \Vert \leq K \bigl\Vert \nabla f(x_{0}) \bigr\Vert \underset{k=0}{ \overset{\infty}{\sum}} \xi^{k} \\ &\leq K \bigl\Vert \nabla f(x_{0}) \bigr\Vert (1-\xi )^{-1}< r. \end{aligned}$$

Thus $x_{n} \in x_{0} +r \mathbb{B}_{ \mathbb{R}^{p}}$ and since $\nabla f(x_{n-1})+B(x_{n-1})(x_{n}-x_{n-1})=0$, from Algorithm $(\mathcal {M}')$ we have

$$\begin{aligned} \bigl\Vert \nabla f(x_{n}) \bigr\Vert &\leq \bigl\Vert \nabla f(x_{n})- \nabla f(x_{n-1})-B(x_{n-1}) (x_{n}-x_{n-1}) \bigr\Vert \leq\varepsilon \Vert x_{n}-x_{n-1} \Vert \\ &\leq\xi^{n} \bigl\Vert \nabla f (x_{0}) \bigr\Vert \end{aligned}$$

and

$$\Vert x_{n+1}-x_{n} \Vert \leq K \xi^{n} \bigl\Vert \nabla f(x_{0}) \bigr\Vert . $$

Since $\xi<1$, the sequence $(x_{n})$ is a Cauchy sequence and hence converges to some $\bar{x}\in\mathbb{R}^{p}$ with $\Vert x_{0}- \bar {x} \Vert < r$. Since ∇*f* is a continuous function, we get $\nabla f (\bar{x})=0$. □

## Conclusions

In this paper, we investigate the concept of first- and second-order approximations to generalize some results such as optimality conditions for a subclass of convex functions called strongly convex functions of order *γ*. We also present an extension of Newton’s method to solve the Euler equation under weak assumptions.
